# Curative intent treatment for pancreatic duct adenocarcinoma invade superior mesenteric vein

**DOI:** 10.1016/j.radcr.2024.09.033

**Published:** 2024-09-24

**Authors:** Ha Pham Hoang, Dung Le Thanh, Khuyen Pham Huu, Thai Pham Quang, An Pham Ba, Duy Ngo Quang

**Affiliations:** a Department of Radiology, Viet Duc Hospital, Hanoi, Vietnam; b Deparment of Radiology, University of Medicine and Pharmacy (VNU-UMP), Vietnam National University, Hanoi, Vietnam; c Department of Radiology, Hanoi Medical University, Hanoi, Vietnam; d Department of Digestive Surgery, Viet Duc University Hospital, Vietnam; e Department of Surgery, University of Medicine and Pharmacy (VNU-UMP), Vietnam National University, Hanoi, Vietnam

**Keywords:** Pancreatic adenocarcinoma, Superior mesenteric vein, Surgical resection

## Abstract

Pancreatic duct adenocarcinoma (PDAC) accounts for about 85-90% of all solid pancreatic tumors, which is well-known for poor prognosis and high morbidity. Despite the massive advent of chemotherapy and radiotherapy in recent years, surgical removal is still considered the cornerstone management option in this situation. Pancreaticoduodenectomy or Whipple procedure is generally contraindicated in metastasis or tumors that encase more than 50% of vessels. Vascular reconstruction is a state-of-the-art technique which requires the remarkable involvement of vascular experts in the setting of PDAC-invading vessels. In this article, we present an exceptional case of a 38-year-old male patient who underwent radical resection for advanced pancreatic cancer with superior mesenteric vein reconstruction by a great saphenous vein.

## Introduction

Local advanced pancreatic cancer (LAPC) refers to the neoplasm that has not spread far beyond the pancreas but has advanced to the point where surgery can not be safely performed due to extensive vascular involvement and a high chance of nonradical resection [1]. In the case of old patients in the advanced stage, biliary stent graft placement and palliative care are frequently applied. Conversely, the long-term outcome of young victims enormously favors the surgical resection method, which can enhance the 5-year survival rate from around 8% to 20% [2]. However, the point of view from the previous century considered the metastatic disease or encasement of major vessels (such as celiac artery, inferior vena cava, or superior mesenteric artery) to be unresectable due to lack of patient benefit evidence while having very high-risk [3]. Nowadays, the development of surgical techniques has allowed the good performance of vascular resection and reconstruction, previously considered contraindications [4]. A minority of high-skill surgeons can even archive R0 resection in a considerable number of patients [5]. The variety of resectability now heavily relies on the level of expertise and willingness of victims to undertake an extended operation. This article presents an exceptional case of a 38-year-old male patient who underwent radical resection for advanced pancreatic cancer with superior mesenteric vein (SMV) reconstruction by saphenous vein. The pathological results confirmed no residual tumor at the surgical margin, and the patient is uneventful after 2 months postoperatively.

## Case report

A 38-year-old male patient came to a private hospital in Vietnam because of vague epigastric pain for several days. He admitted having treatment for acute pancreatitis 2 months previously. His symptoms have been relieved, but the pancreatic enzyme of the latest laboratory test is still as high as 7-8 times compared to the normal index. The patient denied any other medical history of him and his family. No weight loss or fever was documented. The biochemistry blood test revealed a high pancreatic enzyme condition and increased bilirubin level (both direct and indirect). The abdominal computed tomography (CT) demonstrated a 37 × 28 mm poorly enhanced mass at the pancreatic head, which compressed the lower part of the common biliary duct, causing upstream biliary dilation. This mass encased the confluent and distal part of the inferior mesenteric vein. The body and tail of the pancreas were atrophied, and the main duct was dilated due to the mass-effect. Several hepatic hilum and peripancreatic head lymph nodes were detected, the largest is 10 × 15 mm.

**Fig. 1:**
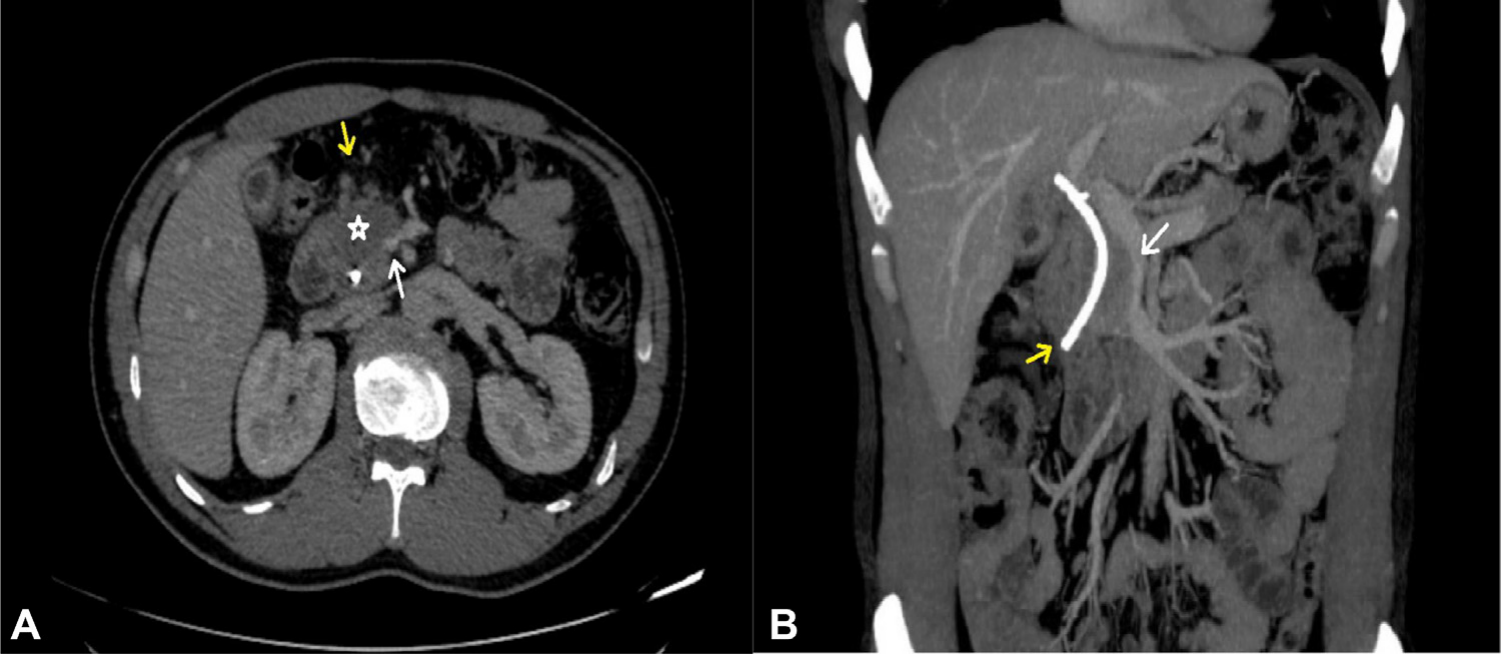
Preoperative abdominal CT scan with contrast enhancement. (A) The axial plane image illustrated the tumor (asterisk) encase the superior mesenteric vein more than 180 degrees (white arrow). The border of this mass with the right colonic mesentery (yellow arrow) and the D2 segment of the duodenum are unclear. (B) The coronal plane image with maximum intensity projection (MIP) reconstruction demonstrated the plastic biliary stent (yellow arrow) and narrowing SMV lumen due to tumoral invasion (white arrow).

Due to his family's wish, he flew to Singapore and had a double-check at a Singapore private hospital. The patient underwent endoscopic retrograde cholangiopancreatography to take biopsy specimens, and the pathological results confirmed pancreatic duct adenocarcinoma (PDAC). Following the consultation with Singapore doctors, a plastic biliary stent graft was placed for symptom relief, and he flew back to Vietnam to undergo a doudenopancreatectomy (DPC) with vascular reconstruction. He was introduced to a gastrointestinal surgery associate professor at Viet Duc University Hospital, Vietnam. The preoperative whole-body CT scan illustrates a plastic biliary stent graft placement with an unclear border with the D2 segment of the duodenum and right colonic mesentery (Fig. 1). There were no abnormal nodes in the liver, lung, or peritoneum. No bone metastasis was detected. Laboratory tests highlighted the elevation of liver enzymes (GOT/GPT: 81/348 U/L) and bilirubin (direct/indirect: 41/37 umol/L). The CA 19-9 was within the normal limit (1.18 U/mL).

**Fig. 2:**
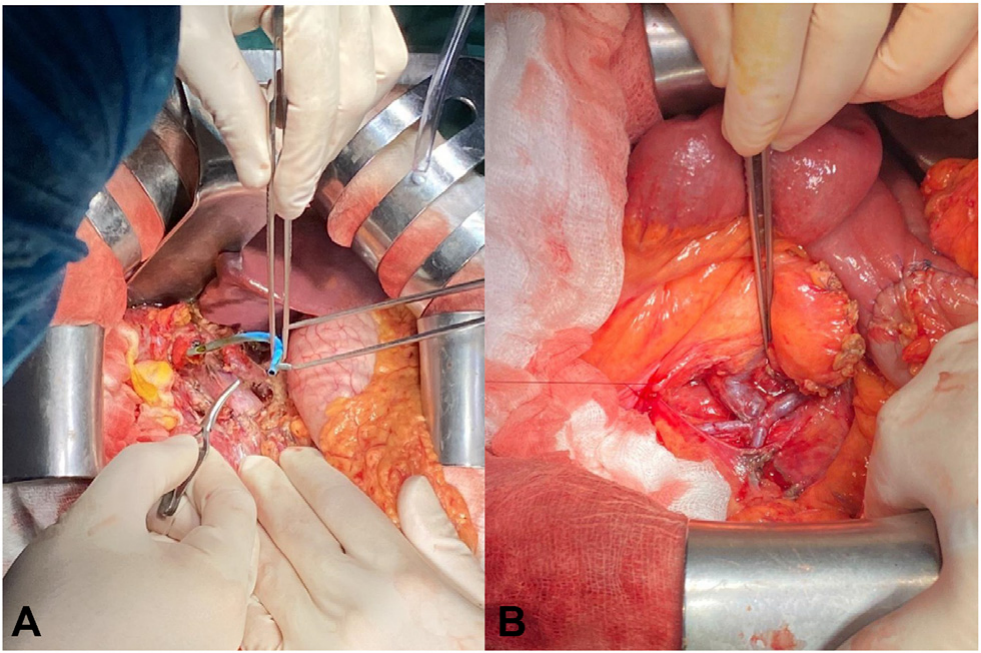
Intraoperative image. (A) The plastic stent was removed from the common bile duct to prepare for resection. (B) The SMV after reconstruction with the anastomosis of collateral venous drainage.

The patient underwent surgical resection several days later. During the operation, the surgeon revealed a firm pancreatic head mass which invaded the lower part of the common bile duct, SMV and right colonic mesentery. The involvement of SMV was measured at 40 mm in length with more than 180 degrees of involvement. The extended pancreaticoduodenectomy was performed with lymphadenectomy. The distal part of SMV was resected and reconstructed using the left saphenous vein (Fig. 2). The surgeon also removed the right colon and mesentery, and a colostomy was opened on the right lower quadrant abdomen. Microscopical pathology results revealed the neoplasm was composed of exocrine pancreatic cells with large eosinophilic nuclei and frequent mitosis. These cells are formed in glandular structures surrounded by remarkably desmoplastic stroma. The final conclusion was that PDAC invaded SMV, duodenum and cecal wall with lymphatic and perineural invasion, 04/13 metastatic lymph nodes (pT4N2Mx) and the surgical margin detected no tumoral cells (R0). Postoperative abdominal CT demonstrated normal flow through reconstructed vessels. The follow-up examination 2 months later was uneventful.

## Discussion

Pancreatic cancer is the seventh cause of death of cancer-related worldwide [6]. Resectable pancreatic cancer accounts for only 15%-20% at the time of diagnosis. The remaining unresectable part contains about 40%-50% of metastatic disease and 30%-40% of LAPC [7,8]. Patients with LAPC rarely underwent surgical resection as a consequence of a high recurrence rate and poor outcome postoperatively. The survival rate of individuals with positive surgical margins is not significantly different compared to nonoperative LAPC with 5-fluorouracil-based chemoradiotherapy [9]. Other better options are biliary drainage, surgical biliary bypass (ie, hepato-jejunostomy), chemotherapy, and local ablative therapy. These methods are particularly suitable for the elderly group and illness conditions. In our case, placing biliary drainage preoperatively in a young patient is due mainly to short-time recanalising the biliary flow purpose. The plastic stent is chosen for temporary use, and the metal material is more feasible for long-term use as the result of a lower rate of complications such as stent occlusion or migration [10]. On the other side of the coin, FOLFIRINOX chemotherapy—well-known induction chemotherapy according to NCCN criteria preferably requires a bilirubin level below 1.5 times the upper limit of normal. Hence, ensuring adequate biliary drainage by stent placing is critical in that scenario.

Induction chemotherapy is chemotherapy performed before surgery or radiation therapy with the goal of improving disease control. FOLFIRINOX (Fluorouracil, leucovorin, irinotecan, and oxaliplatin) therapy has been reported to have a good beneficial effect on improving the survival of selected patients preoperatively [11,12]. Restaging after Chemotherapy commonly requires the assessment of RECIST criteria and CA 19-9 level. The research of van Veldhuisen et al. figured out that a 30% decrease in the level of CA 19-9 can facilitate the sensitivity of resection to 90%, and the elevation of CA 19-9 is a poor prognostic factor [13]. However, our patient showed a level CA 19-9 within the normal limit preoperatively. This fact can be explained by a genetic mutation in the gene for the Lewis antigen (presenting in about 5-10% of people living with pancreatic cancer), which will result in no CA 19-9 produced [14]. Moreover, with the deliberation of pros and cons between induction chemotherapy and the high risk of liver or peritoneal metastasis appearance, we decided to perform surgical resection without preoperative chemotherapy rapidly.

Pancreaticoduodenectomy is often avoided in LAPC because of the perception of complex anatomic structures as morbid and poor long-term prognosis. The unresectability in these cases mainly refers to the extended involvement >180 degrees of major arteries such as celiac or superior mesenteric artery. Arterial resection is only performed in exceedingly rare cases for several challenging reasons. Besides the difficulty of obtaining R0 resection, a secondary decrease in the arterial flow to the liver and stomach enhances the vulnerability to ischemic risk in individuals who underwent major arterial reconstruction [15]. Secondly, the natural coiling of the common hepatic artery has been reported to be helpful in maintaining the hepatic blood flow [16]. Additionally, the problem of arterial reconstruction is also being exploited as the high risk of haemorrhage or disruption of arterial anastomosis due to the effects of a postoperative pancreatic fistula [17].

On the other hand, venous reconstruction seems to have a more favorable outcome. Howard et al. supported the idea that portal venous/superior mesentery (PV/SMV) vein resection can be safely done in the majority of selected patients [18]. The meta-analysis of Song et al. manifested that primary end-to-end anastomosis was the most common venous reconstruction method (68.1%), followed by synthetic vein grafts (16.6%), autologous vein grafts (13.1%), allograft and other materials are less than 3% [19]. Interestingly, the length of venous resection is a valuable prognostic factor. Kaneoka et al. found that portal venous reconstruction (PVR) length less than 3cm results is a good survival rate similar to those without PVR [4]. Conversely, a PVR of more than 3cm results in only 4% of 5-year survival [4]. Furthermore, the existence of collateral venous formation from severe stenosis could also prolong the surgical time. The venovenous bypass technique for PVR could benefit these cases [20]. In our case, the involvement of more than 4 cm SMV is a poor prognostic factor. Generally, the SMV reconstruction is more challenging than PVR as the consequence of numerous collateral branches which require anastomosis after the main SMV is resected. The diversity of choosing autologous vein grafts may vary from study to study. Kaneoka et al. prefer using an external iliac vein to prevent engorgement of the portal system [4]. Leach et al. suggest an internal jugular vein, which is advantageous in restoring venous continuity when the length of PV/SMV resection precludes a primary venovenous reconstruction [9]. The left great saphenous vein was used for our patient, which is the most appropriate segment in length and diameter.

## Conclusion

Surgical resection is the most important treatment method for PDAC. The indication of pancreaticoduodenectomy may vary from patient to patient, depending on the patient's age, the presentation of distal metastasis, and the degrees and lengths of major vessel encasement. Vascular reconstruction is a state-of-the-art technique that can be safely done with high performance in a select number of individuals.

## Ethical statement

Appropriate written informed consent was obtained for the publication of this case report and accompanying images.

## Author contributions

Ha Pham Hoang and Duy Ngo Quang contributed equally to this article as co-first authors. All authors have read the manuscript and agree to the contents.

## Patient consent

Informed consent for patient information to be published in this article was obtained.
